# Rapid Progression to End-Stage Renal Disease in a Young Caucasian Female Newly Diagnosed With HIV Despite a Normal CD4 Count and Viral Load: A Case Report

**DOI:** 10.7759/cureus.86763

**Published:** 2025-06-25

**Authors:** Vikash Kumar, Mona Ghias, Eric Huang, Leonard R Maier, Sharath Rajagopalan

**Affiliations:** 1 Internal Medicine, West Virginia University (WVU) Medicine, Ruby Memorial Hospital, Morgantown, USA; 2 Hospital Medicine, West Virginia University (WVU) Medicine, Ruby Memorial Hospital, Morgantown, USA

**Keywords:** chronic kidney disease (ckd), end stage renal disease (esrd), esrd, hiv, hiv associated nephropathy (hivan)

## Abstract

This is a case of a 31-year-old Caucasian female with a new diagnosis of human immunodeficiency virus (HIV) infection. At the time of diagnosis, her helper T cell (CD4 cell) count was within normal limits, and the viral load was low (356 copies/mL). She initially presented with uncontrolled hypertension and acute kidney injury (AKI) on a background of known chronic kidney disease stage IV (CKD-IV). Renal biopsy during admission revealed collapsing glomerulopathy with IgA deposits, findings consistent with HIV-associated nephropathy (HIVAN). Her disease course progressed quickly to end-stage renal disease (ESRD), requiring initiation of hemodialysis shortly after diagnosis.

## Introduction

Human immunodeficiency virus (HIV)-associated nephropathy (HIVAN) is one of the classic kidney diseases commonly associated with advanced HIV infection. HIVAN usually affects individuals of African ancestry exclusively, with a prevalence ranging from 3 to 12% [1]. Classically, patients develop severe nephrotic syndrome resulting in irreversible renal failure and rapid progression to end-stage renal disease (ESRD) [1]. The incidence and mortality of HIVAN have dramatically declined since the initiation of antiretroviral therapy (ART) [1]. It is characterized histologically by collapsing glomerulopathy and focal segmental glomerulosclerosis (FSGS), with or without mesangial IgA deposits on renal biopsy [1]. Early diagnosis and prompt initiation of ART are crucial to prevent progression to ESRD. HIVAN most often presents in patients with severe immune compromise, typically associated with high viral loads and low CD4 counts, as well as the absence of treatment for their HIV infection. In this case, we present a 31-year-old Caucasian female with newly diagnosed HIV infection who presented with AKI on a background of CKD-IV. After ruling out other causes of nephrotic syndrome, a renal biopsy revealed collapsing glomerulopathy, compatible with HIVAN in the setting of untreated HIV infection, despite a normal CD4 count of 1220 cells/μL and a very low viral load (356 copies/mL).

## Case presentation

The patient is a 31-year-old Caucasian female with a pertinent medical history of hypertension (HTN), chronic kidney disease stage IV (CKD-IV) with a baseline creatinine of 2.5 mg/dL (normal: 0.6-1.1 mg/dL), and a recent diagnosis of HIV infection. She presented to the hospital with hypertensive crisis and reported that she had been diagnosed with HIV several weeks earlier and was still awaiting results for viral load and genotype. She had been following with nephrology intermittently over several months; however, she was lost to follow-up, and a renal biopsy was never completed. In the absence of biopsy, her CKD stage IV was presumed to be caused by reflux nephropathy.

Upon admission, the patient’s laboratory studies showed a significantly elevated creatinine of 9.52 mg/dL (normal: 0.6-1.1 mg/dL), with significant proteinuria, microhematuria, and a protein-to-creatinine ratio of 4475 mg/g (normal: less than 200 mg/g). Abdominal ultrasound demonstrated bilaterally normal kidneys (Table 1).

**Table 1:** Laboratory workup. ANA: antinuclear antibody, ANCA: antineutrophil cytoplasmic antibody, C3: complement component 3, C4: complement component 4, dsDNA: double-stranded deoxyribonucleic acid.

Parameters	Patient Values	Reference Range
Creatinine	9.52 mg/dL	0.6-1.1 mg/dL
Protein/creatinine ratio	4475 mg/g	<200 mg/g
ANA	Negative	Negative
ANCA	Negative	Negative
dsDNA	Negative	Negative
C3	109 mg/dL	81-157 mg/dL
C4	9 mg/dL	<8 mg/dL

Serologic workup for autoimmune and vasculitic etiologies was negative (antinuclear antibody (ANA), antineutrophil cytoplasmic antibody (ANCA), dsDNA, C3, C4).

**Figure 1:**
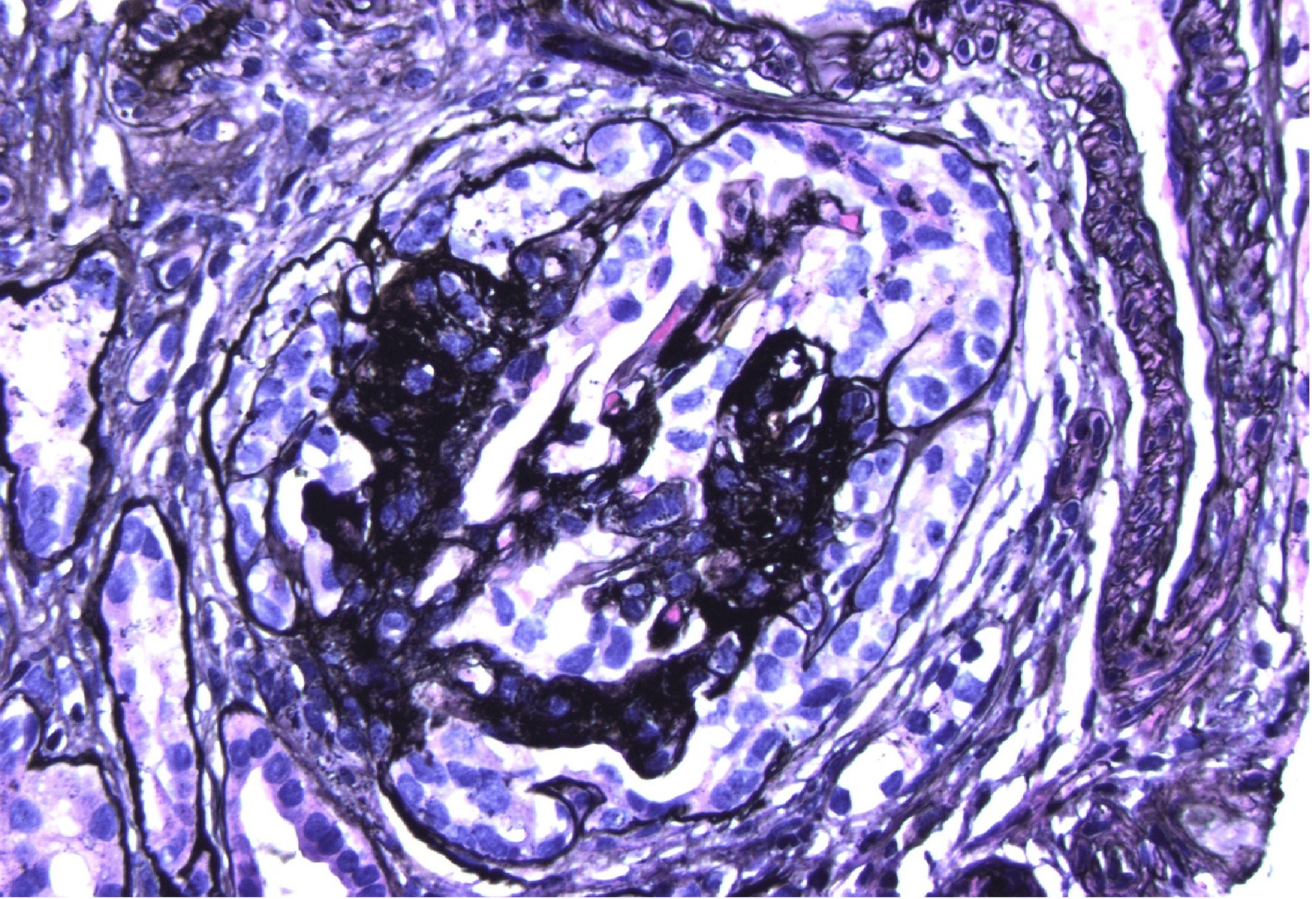
Renal biopsy demonstrating collapsing glomerulopathy. Light microscopy of renal tissue reveals characteristic features of collapsing glomerulopathy. There is global collapse of glomerular capillary tufts with marked hyperplasia and hypertrophy of the overlying podocytes. Extensive glomerulosclerosis, tubular atrophy, and interstitial fibrosis are also noted, which are hallmark findings consistent with HIV-associated nephropathy (HIVAN).

**Figure 2:**
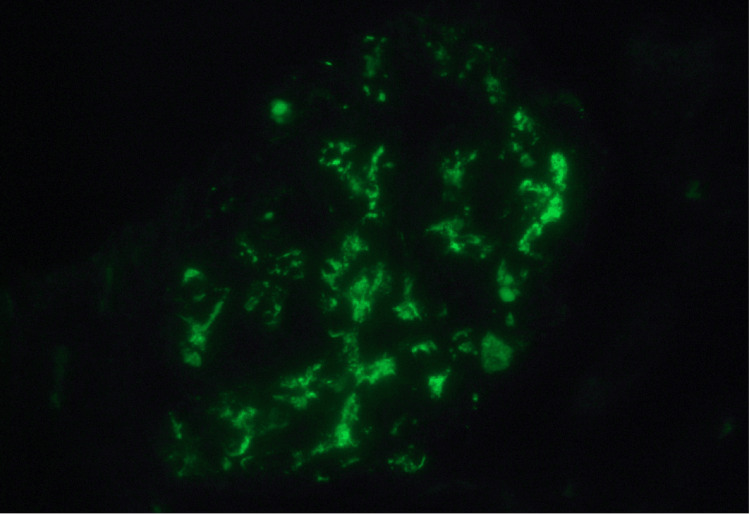
Immunofluorescence microscopy highlighting IgA and complement deposition. Immunofluorescence staining shows intense granular mesangial deposition of immunoglobulin A (IgA, 3+), with mild IgM (1+) deposition.

The patient underwent renal biopsy, which revealed collapsing glomerulopathy with extensive glomerulosclerosis, severe interstitial fibrosis, and tubular atrophy (Figure 1). Immunofluorescence microscopy demonstrated granular mesangial IgA (3+), C3 (3+), and IgM (1+) deposits (Figure 2).

**Figure 3:**
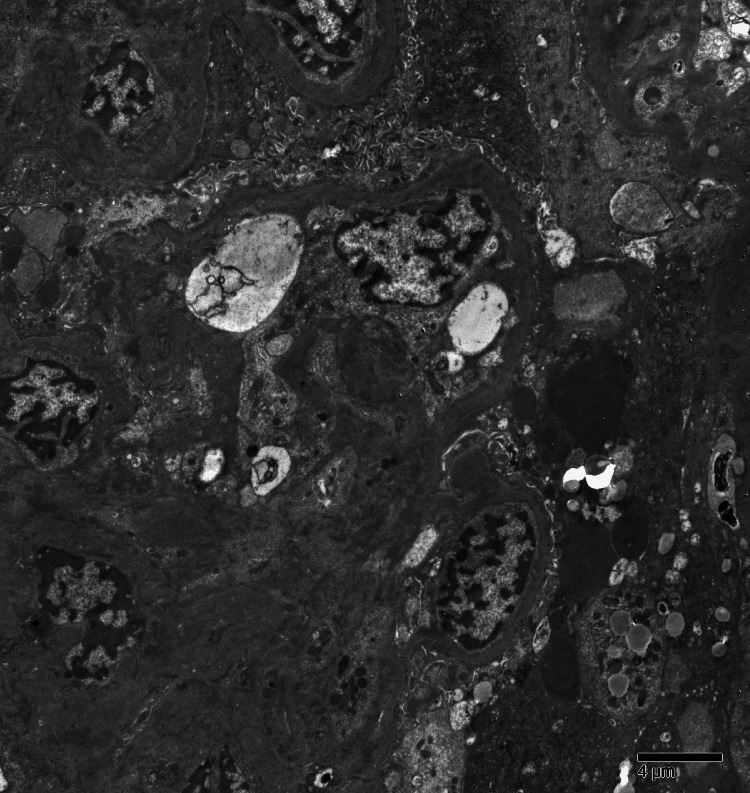
Electron microscopy confirming collapsing glomerulopathy with podocyte injury. Electron microscopy demonstrates widespread effacement of podocyte foot processes and mesangial immune-type electron-dense deposits. These ultrastructural findings are diagnostic of collapsing glomerulopathy, confirming diagnosis of HIV-associated nephropathy (HIVAN).

Electron microscopy confirmed collapsing glomerulopathy with immune-type electron-dense deposits in the mesangial areas and podocyte foot process effacement (Figure 3). These findings were consistent with HIVAN.

After biopsy confirmed HIVAN, she was initiated on ART with dolutegravir and rilpivirine. Unfortunately, due to severe kidney damage, the patient progressed rapidly to ESRD, requiring initiation of inpatient hemodialysis.

She received a tunneled dialysis catheter (TDC) and was eventually transitioned to a regular hemodialysis schedule. Following successful ART initiation and dialysis training, the patient was discharged home and provided follow-up with nephrology, transplant nephrology, and infectious disease specialists.

## Discussion

HIVAN is an aggressive form of collapsing focal segmental glomerulosclerosis [2]. The condition is predominantly seen in African Americans. ART has reduced the incidence of HIVAN; however, it remains the third leading cause of ESRD in African Americans [2]. Typically, HIVAN is seen in HIV-infected patients who have a low CD4 count and high viral load, and about 90% of those cases progressing to ESRD are of African American descent [2]. Our patient’s presentation as a young Caucasian female with normal CD4 count and extremely low viral load highlights the varying and unusual presentations of HIVAN. This case emphasizes the importance of maintaining a high index of suspicion for HIVAN in all patients infected with HIV who are experiencing renal dysfunction and proteinuria, regardless of racial background, CD4 count, or viral load.

Patients with HIVAN typically present with proteinuria, elevated blood urea nitrogen (BUN) and creatinine levels, along with enlarged and echogenic kidneys on ultrasound. A prompt kidney biopsy should be performed [3]. If the diagnosis confirms HIVAN, it will show collapsing focal segmental glomerulosclerosis with microcystic tubular dilatation, along with interstitial inflammation [3]. The hallmark of HIVAN is podocyte proliferation; however, it may not be as pronounced in patients on ART [3].

In patients with HIVAN, early diagnosis and prompt initiation of ART can help prevent or slow the progression of kidney disease to ESRD [4]. Patients can be started on steroids and angiotensin-converting enzyme inhibitors (ACE-I) to slow the progression of renal disease [5]. Even with early detection and treatment, some may have established renal damage that can still lead to ESRD [6].

Studies have shown that a significant number of patients diagnosed with HIVAN have other acquired immunodeficiency syndrome (AIDS)-defining illnesses [6]. This case highlights the need for additional research into the complex and varying presentations of HIVAN in patients who would not meet criteria for diagnosis of it initially.

## Conclusions

HIVAN is a serious complication of HIV infection that can lead to rapid progression to ESRD. This condition is typically seen in patients of African descent and in patients with HIV who either have an opportunistic infection or a CD4 count of less than 200 per cubic millimeter. This highlights the uniqueness of our case and the need to maintain a high index of suspicion. Early diagnosis with renal biopsy is crucial for optimal management, given the rapid progression to ESRD in these patients.
